# Impact of Deoxynivalenol on the Intestinal Microflora of Pigs

**DOI:** 10.3390/ijms10010001

**Published:** 2008-12-27

**Authors:** Yann J. Waché, Charlotte Valat, Gilbert Postollec, Stephanie Bougeard, Christine Burel, Isabelle P. Oswald, Philippe Fravalo

**Affiliations:** 1 French Agency for Food Safety (AFSSA) – Zoopôle des côtes d’Armor, BP53, 22440 Ploufragan – France. E-Mails: y.wache@ploufragan.afssa.fr (Y. W.); g.postollec@ploufragan.afssa.fr (G. P.); s.bougeard@ploufragan.afssa.fr (S. B.); c.burel@ploufragan.afssa.fr (C.B.); p.fravalo@ploufragan.afssa.fr (P. F.); 2 French Institute of Agricultural Research (INRA) – UR 66- Unité de Pharmacologie-Toxicologie, 180 chemin de Tournefeuille, 31027 Toulouse cedex 3 – France. E-Mail: isabelle.oswald@toulouse.inra.fr

**Keywords:** Mycotoxin, Deoxynivalenol, pigs, intestinal microflora, growth performance, faeces, anaerobic sulfite-reducing bacteria, CE-SSCP

## Abstract

Deoxynivalenol (DON), a mycotoxin produced by some *Fusarium* species, is a frequent contaminant of cereal. In the present study, 24 weanling piglets received either control feed or feed naturally contaminated with DON (2.8 mg/kg) for four weeks. Consumption of contaminated feed significantly reduced the animal weight gain during the first week of the experiment, but had a moderate effect on cultivable bacteria in the pig intestine. By contrast, changes in the intestinal microflora were observed by Capillary Electrophoresis Single-Stranded Conformation Polymorphism (CE-SSCP) in DON-exposed animals, suggesting an impact of this toxin on the dynamics of intestinal bacteria communities.

## 1. Introduction

Mycotoxins are fungal secondary metabolites, potentially hazardous to human and animal health following consumption of contaminated food or feed. Contamination of cereals with mycotoxin is a worldwide problem leading to important economic losses for the agricultural industry [1]. The toxicological syndromes caused by ingestion of mycotoxins range from sudden death to reproductive disorders and growth impairment. Consumption of fungal toxins may also decrease resistance to infectious diseases [1, 2].

Deoxynivalenol (DON), also known as vomitoxin, is a mycotoxin of the trichothecene family that is mainly produced by *Fusarium* spp. DON is commonly detected in cereals, particularly in wheat, barley, maize, and their by-products. It is the most prevalent trichothecene contaminant in crop production in Europe and North America [3]. This toxin is resistant to milling, processing and heating, and, therefore, readily enters the human and animal food chains [4]. DON exhibits toxic effects in humans as well as in all animal species investigated to date [5, 6]. Among animal species, pigs show a relative high sensitivity to DON, and, because of the high percentage of wheat in pig diets, swine could be at a greater risk of exposure to this toxin. In swine, the initial adverse effect observed after DON exposure is reduced feed intake. Growth (anorexia and decreased nutritional efficiency), immune function (enhancement and suppression), and reproductive performances (reduced litter size) are also adversely affected by DON [7].

The intestine is the major site of DON absorption [5]. In the pig, DON is rapidly and efficiently absorbed, most probably in the upper part of the small intestine, and is mainly excreted in the urine, with no accumulation in tissues [8, 9]. Consumption of DON-contaminated feed in pigs impacts the gastrointestinal tract, causing epithelial injuries of the stomach and the intestine, leading to intestinal inflammatory response [5, 10, 11]. *In vitro* and *in vivo* studies have also demonstrated that DON inhibits intestinal nutrient absorption [12–14], alters intestinal cell functions [15, 16], and compromises the intestinal barrier function [16, 17].

By contrast, the effect of DON on the intestinal microflora has been poorly investigated. *In vitro* studies have identified intestinal bacterial strains that promote metabolism, binding or detoxification of DON [18–22]. Direct impact of DON on intestinal microflora composition has never been reported and only few data are available for other members of the trichothecene toxin group. Feeding pigs with the T-2 toxin resulted in a substantial increase of aerobic bacterial counts in the intestine [23]. By contrast, feeding pigs with fumonisin was not reported to induce any modification of *Salmonella* counts in the ileum, caecum and colon in an asymptomatic carriage pig model [24] and bacterial growth of species representative of the human intestinal flora was not affected by this mycotoxin [25].

Stability of the intestinal flora appeared to be an important factor for animal health [26, 27]. The aim of the present study was to characterize the effect of chronic exposure due to consumption of naturally DON-contaminated diet on the stabilized intestinal microflora of pigs. The DON effect was assessed by bacterial counting of aerobic and anaerobic cultivable-indicators, relevant for pig health status studies. The dynamic of the total bacterial community was investigated by molecular technique (Capillary Electrophoresis Single Stranded Conformation Polymorphism, CE-SSCP) in order to investigate culture-independent bacterial populations.

## 2. Results

### 2.1. Zootechnical performance

A significant reduction of daily weight gain was observed in piglets exposed to the contaminated diet when compared to animals receiving the control diet (Table 1). This effect was transient and no differences in the daily weight gain of animals feed with control or contaminated diet was observed during the second, third and the fourth week of the experiment (Table 1). During the entire experimental period, no animal manifested diarrheic episodes, vomiting or hypothermia symptoms.

### 2.2. Bacteriological analysis

Faecal samples collected on days 0, 7, 14 and 28, from animals receiving either the DON–contaminated or the control diet and stored frozen were analysed by classical bacteriological analysis. The effect of DON was first investigated by measuring total faecal Aerobic Mesophilic Bacteria (AMB) and Anaerobic Sulfite-Reducing (ASR) bacterial counts representing the dominant cultivable bacterial population. The analysis was completed with a molecular-based method, by comparing the CE-SSCP patterns from dominant population analysis of the individual intestinal flora.

We verified that freezing only had a minor effect on the bacterial count. Indeed, the bacterial load before and after freezing decreased by 0.45 to 1.08 log CFU/g for ASR and by 0.18 to 1.28 log CFU/g for AMB. Thus, the decrease was lower than 1.5 log CFU/g for both bacterial groups.

Total AMB and ASR bacteria were used as an indicator of aerobic, facultative anaerobic and anaerobic flora. Data reported in Table 2 indicated that the number of bacteria (AMB and ASR) in the faecal samples slightly evolved during the experimental period. The number of AMB was significantly increased during the first week of the experiment, reached a plateau (days 7 to 14) and then decreased only in samples from animals receiving the control diet. The concentration of ASR in faeces was stable during the first two weeks of the experimental period and decreased in both groups (control and contaminated diets) at the last sampling date (day 28). At the end of the trial, AMB concentrations were slightly but significantly higher in pigs fed the DON-contaminated diet than in the control group (Table 2).

A molecular analyse was also performed to determine the effect of the DON contaminated diet on faecal microflora balance. The dynamic of the bacterial communities was evaluated by means of several parameters such as the dominance and the richness indexes from CE-SSCP profiles. The dominance index (S), referring to the diversity, was conserved throughout the experiment in both control and DON-exposed groups (Table 3). In the control group, the richness index (R), based on the peak numbers, decreased significantly at day 14 and was lower in the control group when compared to the DON-exposed group (Table 4).

To calculate the similarity between communities, fingerprints are usually analysed by cluster analysis, e.g., unweighted pair-group method based on arithmetic means [28], or by Principal Component Analysis (PCA) statistical analysis. In the present study, cluster analysis and PCA were not performed because samples from each animal taken at different times were considered as dependent data and the number of independent data (different animal) was too small to provide a robust cluster or PCA analysis. The distances between bacterial communities for animals fed control or contaminated diets were investigated by comparing the peak area for all samples according to contrast analysis associated with the linear mixed models.

**Figure 1.**
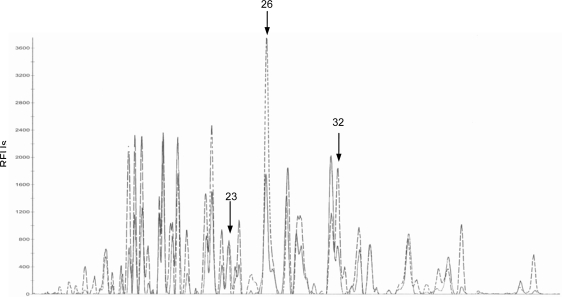
CE-SSCP profiles of faeces from control animals (dotted line) or from DON-exposed animals (continued line) group at day 28. Both profiles were obtained after normalisation with the GeneMapper software (Applied Biosystems, Foster City, USA) and standardisation (aligning). Indicated peaks are those whose area is significantly modified during the whole of the experiment according to contrast analysis associated with the linear mixed model (p<0.05). RFUs, Relative Fluorescence Units.

Based on two repetitions for each sample, the statistical analysis with the linear mixed model did not show any significant differences between samples. In CE-SSCP profiles, among all observed peaks, 3 peaks showing a significant treatment by time interaction were identified according to the linear mixed model (Figure 1; Peak 23, p = 0.024; Peak 26, p = 0.015; Peak 32, p = 0.004).

According to these peaks, the days showing a significant difference of the peak area between animals feed control and DON-contaminated diets were then determined (Table 4). At day 14, the area of peak 23 was 5.2 fold higher (p<0.05) for control animals compared to that of DON-exposed animals. At day 28, the areas of peaks 26 and 32 were lower for control animals compared with those of DON-exposed animals (2.5 fold, p<0.01 and 3.1 fold, p<0.01, respectively).

## 3. Discussion

The main purpose of this study was to evaluate the effect of a diet naturally contaminated with DON on the intestinal microflora of the pig. Indeed, the intestinal microflora is very important because there are close links between the host and its intestinal microflora especially through the immune response and via the metabolic products of fermentation processes. Thus an impaired balance of the intestinal flora could have many adverse effects on the health of the host [29].

One of the challenges of the experimental design was to find raw materials naturally contaminated with DON and not contaminated by other toxins from *Fusarium*. The experimental diet presented a level of DON of 2.8 mg per kg, with low levels of other mycotoxins. This DON contamination level is comparable with that used in other studies [16, 30, 31] and is in accordance with the higher levels currently found in feed manufactured with usual feed constituents.

The main effect of DON ingestion, especially in pigs, is a decrease of both feed intake and weight gains [6, 7]. These effects are observed for contamination level above 1 mg/kg feed and are dependent on the age of animals and the feeding period [7]. In the present study, feeding pigs with a diet naturally contaminated with DON (2.7 mg/kg) had a transient negative effect on the daily weight gain of the animals. A reduced feed consumption was also observed, however, as the feed consumption could only be measured at the pen levels (two pens per diet) and the effect of DON was not statistically significant. These results are in agreement with other studies, showing a transient effect of DON on feed intake in pigs [32, 33]. However, other experiments using DON levels above 3 mg/kg feed, have shown a persistence, for up to eight weeks, of the DON effect on growth parameters [10, 34]. Our results confirmed that DON has an impact on zootechnical parameters in pigs without clinical signs of disease and that DON-contamination could have a more deleterious effect on growth performances than a moderate feed restriction.

More recently, several studies have investigated the interaction between some intestinal microflora strains and mycotoxins, such as Trichothecene, in *in vitro* experiments [18–20, 22, 35]. These studies aim to identify bacterial strains that could reduce the toxicological effects of mycotoxins. To our knowledge, few studies have investigated the effect of Trichothecene on the intestinal microflora [23]. After administration of T-2 toxin at a dose of 5 mg/kg, an increase in bacterial counts in the gut of rat and pigs, especially of Coliform bacteria, has been observed. The authors related this modification to a change in the host’s natural resistance of the intestinal content [23].

In the present study, although a slight but statistically significant, effect of DON, being lower than 1 log CFU/g, was observed on AMB concentration at day 28, no significant effect of DON could be observed using the culture-dependent method on the ASR concentration mainly represented by the *Clostridium* group, representing the dominant cultivable intestinal bacterial population [36]. Nevertheless, changes in AMB and ASR counts in faeces of pigs were observed in relation to the age of the pigs, may correspond to the adaptation to experimental diet [37]. Indeed, the percentage of wheat in the diet was progressively increased before experimental diet introduction. Comparison with intestinal bacterial counts performed in other studies [38, 39] is difficult because of the variability between experimental designs. Moreover, we should be keep in mind that estimates of culturability of bacteria in the gastrointestinal tract vary from 10 to 50% [40] and classical bacteriological counts cannot illustrate the changes in individual species abundance of the microbial community.

Based on the electrophoretic separation of single stranded DNA fragments according to size and secondary structure, the CE-SSCP technique provides fingerprints of a complex microbial community [41, 42]. This method was used in order to assess the potential changes of bacterial community in animals fed either control or DON-contaminated diets. These changes were observed by calculating the dominance index, reflecting the diversity (Simpson index, S) and the richness (R) index, according to the peak area and the number of peaks, respectively. No effect of DON on the diversity degree was observed. The diversity is conserved from day 0 to day 28, corresponding to 9 to 13 week-old pigs, in both control and DON-exposed groups. These results are in accordance with previous reports showing that the normal adult flora is developed and becomes stable and characteristic for each animal after 4 to 6 weeks post weaning [43, 44]. By contrast, the richness index evolved during the experiment, being lower in the control group than in the DON-exposed group at day 14. However, this difference seems to be related more to the decrease of the R index in the control group than to a potential effect of DON.

The CE-SSCP is able to show changes in total intestinal microflora, although a common criticism of community fingerprinting techniques is the potential for artifacts. The number of peaks allows an estimation of the species richness, although in a complex matrix it may only represent the dominant populations. In theory, one bacterial species is represented by one peak. However, because of intraspecies operon heterogeneity or potential multiple conformation of the same sequence, it has been observed that one organism may yield more than one band or peak [45, 46]. The extraction and the number of PCR products can be biased to specific groups [47] or overestimated because of multiple rDNA copies per genome [48]. In addition, when more than 35 peaks are present, the number of peaks does not directly correlate with the degree of diversity [49].

Despite the high variability between individual animals and the lack of significant changes due to by the effect of DON on the diversity of the bacterial community, the statistic linear mixed model developed in this study allowed the identification of specific peaks, which presented significant area variations, representing specific changes in CE-SSCP patterns. To our knowledge, this is the first time that an impact of DON on the intestinal microflora has been demonstrated. Thus, as this effect could have consequences for the pig health status, further investigations would be interesting. Although the most important changes in microflora are usually observed just after the weaning when the most digestive problems occur, the variability of intestinal microflora is very high during this period. As the high variability could have masked some changes, as a consequence this first study was performed on 9-week-old pigs when the microflora is stabilised [43, 44] and when the diet formulation is constant. Further studies on the influence of DON on young animals could be relevant.

The microflora in the ileum is distinct in composition from that in the caecum, colon or faeces [50], knowledge of the effect of mycotoxin on microbial intestinal flora being limited. Only faecal samples were analysed in this explorative study. The results of this first study clearly demonstrate that low doses of DON which could be usually find in livestock animals feedstuff had an impact on the faecal flora. Considering the faecal flora, these original findings are very interesting because it hypothesized that low doses of DON could modify the gastrointestinal associated microflora. Future studies on the impact of DON on different parts of the gastrointestinal tract, especially the ileum where most DON is absorbed [8, 9] and the large intestine where DON is metabolised [9], will help to better define the impact of DON of intestinal microflora. Other doses of mycotoxin could be tested in order to evaluate the impact of the acute toxicity of mycotoxin on pig microflora.

The statistical model developed in the present study could be of interest to identify slight changes, which cannot be identified by classical statistical analysis. However, further investigations are needed to identify and sequence the genome of the bacterial communities corresponding to the selected peaks. To identify peaks of CE-SSCP runs, the microbial community PCR products have to be analysed in parallel with PCR products obtained from a clone library of the selected sample, and the average and standard deviation of the relative migration distance of all peaks could be determined. CE-SSCP is a promising high-throughput tool for profiling complex communities [51]. Despite the extensive use of CE-SSCP, the assignation of peaks remains fastidious and not always possible in particular in a complex matrix. Other fingerprint methods on gels (SSCP, Denaturing Gradient Gel Electrophoresis DGGE) or by chromatography (Denaturing High Performance Liquid Chromatography DHPLC) allow the assignation of bands or peaks by sequencing directly from the gel or eluted samples. Nevertheless, due to artefacts, the identification of unique species by band or peak is infrequent in a complex matrix. It would also be interesting to ascertain if these changes in the intestinal microflora are dependent on the dose of DON ingested and if they would persist when pigs return to a mycotoxin-free diet.

## 4. Experimental Section

### 4.1. Animals, housing and experimental design

Animals were used in the accordance with Guidelines National Institutes of Health Guide and the French Ministry of Agriculture for the care and use of laboratory animals. The study was carried out with twenty-four 9-week-old specific pathogen-free Large White pigs, born at the AFSSA experimental farm at Ploufragan, France. After weaning at four weeks of age, animals were individually identified and divided into two groups (6 females and 6 castrated males per group), with a mean initial live weight of 30.0 ± 2.1 kg and 29.6 ± 1.6 kg respectively. At nine weeks of age, pigs were fed with the control or the experimentally contaminated diet, which constituted the day 0 of the trial. Each group, divided into two pens of six animals, was housed in a separate block of the housing unit with free access to feed and water. Pigs were randomly distributed within pens in order to avoid the effect of the pen and of the lineage. Pigs were examined daily for body temperature and faeces aspect. No morbidity or mortality was recorded during the study. Room temperature and air velocity were automatically controlled, and pens were daily cleaned. Feed refusals were weighed and discarded. In each group, six pigs (three per pens) were sacrificed at day 7; the other six animals were sacrificed at the end of the experiment (at day 28).

### 4.2. Experimental diet

Pigs were fed *ad libitum* a based diet prepared locally and formulated according to energy and dietary amino acid requirements of growing pigs [52]. Two different batches of wheat were used in the diets, one control batch was free of mycotoxin-contamination and one batch was naturally contaminated with DON (Table 5). Type A trichothecenes (diacetoxyscirpenol, T-2 toxin, HT-2 toxin and 15-acetoxyscirpenol), type B trichothecenes (DON, 3-acetyl DON, 15-acetyl DON and nivalenol), Zearalenone (ZEA) and ochratoxin were analysed in the final diet. Mycotoxins were analysed using Liquid Chromatography coupled with tandem Mass Spectrometry (LC-MS/MS) techniques and their detection limit was 10 μg/kg feed (Laboratory LDA 22, Ploufragan, France). Only mycotoxins which were detected at least in one of both experimental diets are presented in Table 5.

### 4.3. Zootechnical performances and sample collection

Pigs were weighted weekly. The daily weight gain was calculated per animal and feed intake was measured per pen, per week during the four weeks of the experiment. Feed conversion ratio was calculated by week per pen on the basis of the consumption.

Faecal samples were individually collected on day 0 (before DON exposure), and days 7, 14, and 28 of the experiment. Samples were immediately divided into two fractions and stored at −20°C: one fraction was used to count intestinal bacterial populations by cultural methods (see bacteriological counts), the other being used to perform molecular analysis by Capillary Single-Stranded Conformation Polymorphism (CE-SSCP).

### 4.4. Bacteriological counts

*Media:* Tryptone salt tubes (ref. 42076) were obtained from Biomerieux (Craponne, France), Tryptone sulfite-cycloserine (TSC, AEB152892), and peptone water (AEB140302) were purchased from AES Chemunex company (Bruz, France). Tryptone soya agar (TSA, CM0131) was obtained from Oxoid (Dardilly, France). The D-Cycloserin antibiotic was purchased from Sigma-Aldrich (St Louis, USA).

*Bacterial culture:* Due to experimental limitations, analyses could not be performed on fresh samples. Hence, individual samples were frozen at −20 °C. In order to control the effect of freezing on bacterial counts, at each sampling day, a sample (15 g) obtained from a pool of fresh faeces from six control animals was analysed by plate counting before and after freezing. Individual and pool samples were thawed in ice for 1 hour before plating. Fifteen grams of thawed faeces were poured in buffered peptone water at 1:10 (w/v). The samples were serially 10-fold diluted and 3 dilutions of each sample were plated. Two intestinal bacterial populations were investigated: Anaerobic Sulfite-Reducing bacteria (ASR) and Aerobic Mesophilic Bacteria (AMB). AMB colonies were counted using a Spiral^®^ DS Plus plater (Interscience, St Nom-La-Breteche, France) after 48 h of growth on TSA plates (30 °C). ASR colonies were counted on plates after 24 h of growth in TSC (37 °C) in sealed jars using the AnaeroGen^®^ (Oxoid, Dardilly, France) system to create an oxygen-reduced atmosphere. The concentration of bacteria in the original sample was calculated as the mean of three dilutions for each faecal sample. The results are expressed as the mean of CFU/g from six animals ± Standard Error (SE).

### 4.5. Capillary Single-Stranded Conformation Polymorphism (CE-SSCP) analysis

Intestinal flora evolution was evaluated by comparison of 48 CE-SSCP profiles from 12 individual faecal samples.

*DNA extraction:* Two hundred mg of thawed faeces were diluted in 1.4 mL of lysis buffer (QIAamp^®^ DNA stool mini-kit, Qiagen, France) and vortexed until completely dissolved. DNA extraction was carried out by using the QIAamp^®^ DNA stool mini-kit according to the manufacturer instructions. Extracted DNA was loaded on 1% agarose gel in order to verify its quality.

*PCR amplification:* For the total microflora analysis, DNA was amplified from 1 μL of extracted DNA solution, and added to the PCR mixture containing 1.3 μL of dNTP (Stratagene, France) (10mM), 5μL of buffer 10X, 1.3 μL of each primer (100 ng/μL), 0.5 μL of *pfu* turbo DNA polymerase (Stratagene, France) (2.5 U/μL), and 40.9 μL of high-purified water. The targeting DNA sequence of the V3 region of DNA 16S was used for total bacteria recognition with already published primers [53] w49 (AGGTCCAGACTCCTACGGG) and w104* (*TTACCGCGGCTGCTGGCAC) (Sigma-Proligo, UK). Primer w104* was labelled with 5’-6 Fam fluorescent dye. Primer w49 was labelled with Hex fluorescent dye. These primers are specific for the Eubacteria phylogenic domain. The mix was run for 2 min at 94°C, then 25 cycles of 30 sec at 94°C, 30 sec at 61°C, 30 sec at 72°C, and 10 min at 72°C. PCR reactions were performed with a Gene Amp 9700 or 2400 (Applied Biosystems, France). After amplification, 10 μL of the amplified product were run on a horizontal 2% agarose gel in TBE 1X, with a DNA ladder of 100 bp size in order to control PCR reactions (Ozyme, France). Gels were stained with Ethidium Bromide (0.5 μg/mL) during 20 min and the images were captured under U.V. illumination by a video system.

*CE-SSCP electrophoresis:* Each DNA from PCR product from was diluted in water according to the intensity signal observed on the agarose gel to obtained standardised samples. 1 μL of the standardised proportion of PCR product was diluted in high purified water (1:5, v:v) and then mixed with 18.5 μL of sequencing buffer mix and 0.5 μL of internal standard HD 400 [rox] (Applied Biosystems, Foster City, USA). After a denaturing step at 95°C during 5 min, the mix was quickly cooled on ice during 10 min before capillary electrophoresis conducted in a Genetic Analyser 3100-Avent (Applied Biosystems, France).

The CE-SSCP gel composition was 6.22 g of CAP polymer (Applied Biosystem, Foster City, USA), 1 g of glycerol (In Vitrogen, France), 1 mL of 10X buffer (Applied Biosystem, Foster City, USA), completed to 10 mL with Milli-Q water. Capillary Electrophoresis was run at 32°C under 15kV. Each 48 PCR products were analysed by 2 CE-SSCP runs. The statistical analysis by the mixed linear model (see below) was performed on duplicate CE-SSCP profiles of all the 48 samples.

### 4.6. Data analysis

All data analyses were computed with the SAS software (SAS, 2004).

*Zootechnical data:* The zootechnical data are the mean ± Standard Error (SE) of results obtained from twelve or six animals, depending on the date of sacrifice of the animal. These data were analysed by ANOVA test for repeated and correlated series, after checking for the homogeneity of the residual variance (Hartley’s test).

#### Bacteriological data

*Bacteriological counts.* Bacteriological count data are expressed as the mean of log (CFU/g) ± Standard Error (SE) from six animals. Statistical analyses were performed with the linear mixed model (see below).

*CE-SSCP.* The SSCP fingerprints were analysed with the GeneMapper^™^ software (Applied Biosystem, Foster City, USA) with a minimum peak height threshold of 50 Relative Units of Fluorescence (RFUs). Profiles obtained were stacked according to the internal standard position. To compare CE-SSCP patterns, additional alignments were done in order to adjust the peak positions of all standardized profiles, using a “profile reference” having the highest number of detectable peaks. The peaks were selected according to contrast analysis associated with linear mixed models.

*Diversity and Richness Indexes.* The Simpson index of dominance, S = 1 – ΣPi^2^, where Pi is the proportion of the peaks area i (a_i_) in CE-SSCP profiles (Pi = a_i_ / Σ a_i_). The S index measures the probability that two individuals randomly selected from a sample will belong to the same species. The diversity decreases when the dominance increases. The Richness R was estimated as the number of peaks present in a profile divided by the maximum number of peaks detectable in a profile (44 peaks) for CE-SSCP patterns obtained from dominant population analysis [49].

*Statistical models:* Linear mixed models were computed with the MIXED procedure of the SAS software [54]. Random and repeated effects were used for both bacterial count and CE-SSCP statistical analysis [55–58]. Treatments by time interaction are introduced into the model as fixed effects in accordance with the design of the study. Animals were incorporated into the model as a repeated measurement factor in order to take into account the within-animal covariability. Finally, the intercept of the model was incorporated as a random effect using an unstructured covariance matrix. In the case of a significant treatment by time interaction, an alternative to multiple comparison procedures, that are not available with linear mixed models, was the use of a limited number of contrasts to test hypotheses of interest. Contrasts were defined to compare both conditions for each time. The repeatability between 2 CE-SSCP runs was included in the linear mixed model and p values between 2 repetitions were determined.

## 5. Conclusions

The mechanism of action of DON on eukaryotic cells is well documented but the effect of this toxin on the intestinal microbiota is largely unknown. In the present study, analysing the CE-SSCP profile of faecal flora, we demonstrate for the first time that DON modifies the intestinal microbiota of the animals. Further analyses are needed to better identify the observed changes.

## Figures and Tables

**Table 1.** Growth parameters of pigs during the experimental period.

Parameters	Experimental Group	Experimental period (in days, d)				
		d-7 to d0(1)	d0 to d7	d7 to d14	d14 to d21	d21 to d28
Daily Weight Gain	Control	0.93 ± 0.05 (2)	0.96 ± 0.04	1.02 ± 0.04	1.15 ± 0.07	1.00 ± 0.12
	DON	0.85 ± 0.04	0.42 ± 0.06 ^***^	0.96 ± 0.06	1.00 ± 0.07	1.07 ± 0.06
Feed intake	Control	1.67 ± 0.05 (3)	2.09 ± 0.09	2.59 ± 0.04	2.85 ± 0.01	3.05 ± 0.04
	DON	1.55 ± 0.04	1.12 ± 0.02	2.77 ± 0.12	2.86 ± 0.01	2.78 ± 0.06
Feed conversion ratio	Control	1.79 ± 0.07 (4)	2.18 ± 0.07	2.54 ± 0.09	2.47 ± 0.02	3.06 ± 0.58
	DON	1.84 ± 0.02	2.68 ± 0.46	2.87 ± 0.11	2.88 ± 0.37	2.60 ± 0.07

^(1)^ Before DON exposure;

^(2)^ Data are mean ± SE of twelve (d-7 to d0), or six (others periods) pigs per group;

^(3)^ Data are mean ± SE of consumption per animal per day, calculated on the basis of collected values in two pens per group (n=2);

^(4)^ Data are mean ± SE, calculated for the period on the basis of collected values in two pens per group; DON-treated and control animals were compared by Student t test:

*** indicates statistically significant differences, *P*<0.001.

**Table 2.** Evolution of bacteria counts in faeces from pigs during the experimental period.

Bacteria	Experimental group	Sampling days (d)			
		d0(1)	d7	d14	d28
Aerobic Mesophilic Bacteria (AMB)	Control	5.24 ± 0.25 ^a^	7.68 ± 0.22 ^b^	7.37 ± 0.13 ^b^	6.53 ± 0.14 ^c^
	DON	5.27 ± 0.08 ^a^	7.37 ± 0.32 ^b^	7.71 ± 0.35 ^b^	7.22 ± 0.10 ^b^
Anaerobic Sulfite-Reducing bacteria (ASR)	Control	9.52 ± 0.05 ^a^	9.31 ± 0.15 ^a^	9.02 ± 0.17 ^a^	7.83 ± 0.16 ^b^
	DON	9.05 ± 0.39 ^a^	8.66 ± 0.38 ^a^	8.45 ± 0.22 ^a^	7.27 ± 0.09 ^b^

Results are expressed as log (Colony Forming Unit/g faeces), mean ± SE (n=6 individual samples);

^a–c^ Data not sharing a common letter within the same bacterial group are significantly different (p<0.05), compared with contrast associated with the linear mixed model, p< 0.05;

^(1)^ before DON exposure.

**Table 3.** Dominance and richness indexes from CE-SSCP patterns.

Index(2)	Experimental group	Sampling days (d)			
		d0(1)	d7	d14	d28
Dominance (S)	Control	0.93 ± 0.01 ^a^	0.92 ± 0.01 ^a^	0.94 ± 0.01 ^a^	0.93 ± 0.02 ^a^
	DON	0.92 ± 0.01 ^a^	0.93 ± 0.01 ^a^	0.94 ± 0.01 ^a^	0.95 ± 0.01 ^a^
Richness (R)	Control	0.86 ± 0.04 ^a^	0.83 ± 0.03 ^a^	0.70 ± 0.02 b*	0.84 ± 0.04 ^a^
	DON	0.84 ± 0.03 ^a^	0.80 ± 0.04 ^a^	0.87 ± 0.03 ^a^	0.83 ± 0.03 ^a^

*Note:* Results are expressed as mean ± SE (n=6 individual samples).

^(1)^ Before DON exposure.

^(2)^ Dominance index (S: Simpson index); Richness index (R).

^a–b^ Data not sharing a common letter within the same row group are significantly different (p<0.05). DON treated and control groups were compared with contrast associated with linear mixed model the linear mixed model;

*, P<0.05.

**Table 4.** Effect of time and diet on the area of selected peaks obtained from CE-SSCP profile analysis.

Peak	Experimental group	Sampling days (d)			
		d0(1)	d7	d14	d28
P23	Control	3.56 ± 0.99 ^a, c^	3.43 ± 2.28 ^a, b^	1.20 ± 0.46 ^b^*	5.12 ± 1.52 ^c^
	DON	1.65 ± 0.32 ^a^	3.96 ± 1.12 ^b^	6.26 ± 1.41 ^b^	5.80 ± 1.29 ^b^
P26	Control	30.80 ± 9.21 ^a^	35.34 ± 4.83 ^a^	7.33 ± 2.65 ^b^	27.44 ± 6.20 ^a^*
	DON	21.21 ± 5.28 ^a, b^	26.27 ± 4.05 ^a^	2.60 ± 0.49 ^b^	10.90 ± 2.71 ^c^
P32	Control	17.18 ± 4.43 ^a^	19.38 ± 2.14 ^a^	4.27 ± 2.55 ^b^	16.28 ± 3.78 ^a^**
	DON	11.43 ± 2.62 ^a^	15.12 ± 2.35 ^a^	8.35 ± 2.67 ^a^	5.21 ± 1.86 ^a^

*Note:* Results are expressed as mean arbitrary units ± SE (n=6).

^(1)^ Before DON exposure.

^a–c^ Data not sharing a common letter within the same row are significantly different (p<0.05). For each peak and at each day, values were obtained for control and DON-treated animals compared with contrast associated with the linear mixed model:

*: p<0.05;

** p< 0.01.

**Table 5.** Ingredient, nutrient composition and mycotoxin contamination of the experimental diets.

	Diet	
	Control	Contaminated
**Energy**		
Net energy (MJ.kg^–1^)	9.6	9.6
**Composition (%)**		
CleanWheat	65	0
Contaminated wheat	0	65
Soybean meal	19.5	19.5
Barley	8.5	8.5
Lucerne	3	3
Calcium phosphorus	1.25	1.25
Clay	1	1
Iodised salt	0.3	0.3
Limestone	0.2	0.2
Additives	0.08	0.08
Vitamins and mineral mixture^1^	1.07	1.07
**Nutrient component (%)**		
Dry matter	87.0	87.2
Mineral matter	6.2	6.1
Brut organic matter	80.9	81.1
Crude fat	3.1	3.2
Nitrogen extract	18.2	18.3
Cellulose	2.8	3.2
Amidon	43.4	42.7
**Mycotoxin (μg.kg^–1^)**(2)		
Deoxynivalenol (DON)	65	2700
15-acetyl DON	nd (3)	20
Nivalenol	nd	75
Zearalenone	nd	275

^(1)^ Provided per kilogram of diet: vitamin A, 13,910 IU (IU, International Unit); vitamin D3, 5,350 IU; vitamin E, 26.8 IU; vitamin B1, 2.5 IU; vitamin K3, 4.3 IU; vitamin C, 21.4 IU; copper sulphate, 16 mg; iron carbonate, 69.5 mg; manganese, 96.3 mg; zinc, 91 mg.

^(2)^ Only mycotoxins detected in at least in one of the experimental diets are mentioned.

^(3)^ nd = not detectable
